# Association between boarding in the emergency department and in-hospital mortality: A systematic review

**DOI:** 10.1371/journal.pone.0231253

**Published:** 2020-04-15

**Authors:** Zoubir Boudi, Dominique Lauque, Mohamed Alsabri, Linda Östlundh, Churchill Oneyji, Anna Khalemsky, Carlos Lojo Rial, Shan W. Liu, Carlos A. Camargo, Elhadi Aburawi, Martin Moeckel, Anna Slagman, Michael Christ, Adam Singer, Karim Tazarourte, Niels K. Rathlev, Shamai A. Grossman, Abdelouahab Bellou

**Affiliations:** 1 Emergency Medicine Department, Dr Sulaiman Alhabib Hospital, Dubai, UAE; 2 Emergency Medicine Department, Beth Israel Deaconess Medical Center, Teaching Hospital of Harvard Medical School, Harvard Medical School, Boston, Massachusetts, United States of America; 3 Emergency Medicine Department, Purpan Hospital and Toulouse III University, Toulouse, France; 4 The National Medical Library, College of Medicine and Health Sciences, UAE University, Al Ain, UAE; 5 Hadassah Academic College, Jerusalem, Israel; 6 Emergency Medicine Department, St. Thomas’ Hospital, London, England, United Kingdom; 7 Department of Emergency Medicine, Massachusetts General Hospital, Harvard Medical School, Boston, Massachusetts, United States of America; 8 Department of Paediatrics, College of Medicine and Health Sciences, UAE University, Al Ain, UAE; 9 Division of Emergency and Acute Medicine, Campus Virchow Klinikum and Charité Campus Mitte, Charité Universitätsmedizin Berlin, Germany; 10 Emergency Department, Lucerne, Switzerland; 11 Department of Emergency Medicine, Stony Brook University, Stony Brook, New York, United States of America; 12 Department of Emergency Medicine, University Hospital, Hospices Civils, Lyon, France; 13 Department of Emergency Medicine, University of Massachusetts Medical School, Baystate, Springfield, United States of America; 14 Global HealthCare Network & Research Innovation Institute LLC, Brookline, Massachusetts, United States of America; Azienda Ospedaliero Universitaria Careggi, ITALY

## Abstract

**Importance:**

Boarding in the emergency department (ED) is a critical indicator of quality of care for hospitals. It is defined as the time between the admission decision and departure from the ED. As a result of boarding, patients stay in the ED until inpatient beds are available; moreover, boarding is associated with various adverse events.

**Study objective:**

The objective of our systematic review was to determine whether ED boarding (EDB) time is associated with in-hospital mortality (IHM).

**Methods:**

A systematic search was conducted in academic databases to identify relevant studies. Medline, PubMed, Scopus, Embase, Cochrane, Web of Science, Cochrane, CINAHL and PsychInfo were searched. We included all peer-reviewed published studies from all previous years until November 2018. Studies performed in the ED and focused on the association between EDB and IHM as the primary objective were included. Extracted data included study characteristics, prognostic factors, outcomes, and IHM. A search update in PubMed was performed in May 2019 to ensure the inclusion of recent studies before publishing.

**Results:**

From the initial 4,321 references found through the systematic search, the manual screening of reference lists and the updated search in PubMed, a total of 12 studies were identified as eligible for a descriptive analysis. Overall, six studies found an association between EDB and IHM, while five studies showed no association. The last remaining study included both ICU and non-ICU subgroups and showed conflicting results, with a positive association for non-ICU patients but no association for ICU patients. Overall, a tendency toward an association between EDB and IHM using the pool random effect was observed.

**Conclusion:**

Our systematic review did not find a strong evidence for the association between ED boarding and IHM but there is a tendency toward this association. Further well-controlled, international multicenter studies are needed to demonstrate whether this association exists and whether there is a specific EDB time cut-off that results in increased IHM.

## Introduction

Emergency department (ED) crowding is one of the most important challenges that hospitals face, and its importance has been increasingly addressed by media and policy makers. Recently, Institute of Medicine identified ED crowding as an important challenge for public health [1].

Literature about ED crowding has grown exponentially and few studies showed that ED crowding increases the length of stay, in-hospital mortality, and critical care unit admission [2,3], and delays to specific treatment for life-threatening diseases such as myocardial infarction [4,5] and community-acquired pneumonia [6]. Crowding is a risk factor for poor quality of care and adverse events after ED evaluation [7,8].

ED boarding (EDB) is one of the most important factor of ED Crowding. It is defined as the patients stay in the ED after the admission was accepted in the hospital, because of absence of inpatient beds [9]. Although, ED crowding was initially blamed on ‘‘unnecessary ED visits” [10–12] and other factors such as delays in laboratory and radiology results and increases in ED visits, recent studies have established that EDB is the most important cause of ED crowding [13–15]. This was highlighted by the American College of Physicians (ACEP), which stated clearly that “*ED boarding is the primary cause of ED crowding”* [9]. Thus, a Task Force was established in August 2007 by ACEP to propose boarding solutions. The task force report, “Emergency Department Crowding: High-Impact Solutions,” was published in April 2008 with the objective to improve in-patient care and to avoid the boarding [9]. ED crowding is also a challenge for the majority of EDs in the world; in the U.S., ED boarding remains the most important factor involved in ED crowding worldwide [16–18].

Boarders are vulnerable because they do not receive the needed care that they would receive in the wards [13]. Several studies showed that EDB increases ventilator-associated pneumonia [19], critical care unit mortality [2], and overall admission [20,21]. It was also observed preventable adverse events and increased medication errors [22,23].

EDB seems to be an important factor in the healthcare system and is associated with multiple undesirable patient outcomes and dangerous events. However, no systematic literature review to date has analysed the relationship between EDB and in-hospital mortality (IHM). Thus, we investigated the impact of EDB on patient outcomes, focusing on the association between EDB and IHM. We conducted a systematic review of published peer-reviewed studies investigating this association.

## Materials and methods

### Eligibility criteria

Articles that were extracted in this review focused on the association between EDB and IHM as the primary objective. Studies analyzing the effects of other factors such as ED crowding or length of stay (LOS) on mortality, as well as health economic studies, were also included if EDB was reported. Reviews and editorials were not included but they were screened and their reference lists were reviewed for relevant studies. We included studies performed in the ED and excluded those performed only in urgent care, primary care, or ICU settings. We included studies performed in urban and community hospitals, public and private EDs, academic and non-academic EDs, level I, II and III trauma centers, as well as studies analyzing the general population presenting to the ED. Studies with a focus on specific adult ED populations were also considered (e.g., geriatric or psychiatric patients; patients with acute myocardial infarction, pulmonary infection, trauma). To facilitate comparison between studies, publications on pediatric emergency care (patients aged less than 18 years) were not included. Our search included articles reported in English as well as publications in other languages. Studies selected for inclusion analyzed IHM following admission from the ED regardless of timing. Studies that analyzed mortality in the ED and following ED presentation were also included.

### Predictors (exposures) and outcomes

We used the classical definition of boarding, defined as the time that patients spent in the ED after an admission decision was made until they left the ED for admission. There is no international definition of a boarding cut-off that increase IHM, we screened all studies regardless of the boarding time (BT) cut-off. BT includes three successive time intervals: the time to decision to admit, time to admission order, and time to ED exit. In addition, we considered interventions aiming to decrease BT when authors analyzed the impact of these interventions on mortality. All admitted ED’ patients where included. The primary outcome of the eligible studies was IHM among patients admitted from the ED. We considered all-cause mortality and mortality related to specific conditions. When IHM data were unavailable, early mortality rates were analyzed at the time point(s) reported in the study.

### Information sources and search strategy

An extensive search was realized by a medical librarian specialized in systematic reviews (LÖ) in close collaboration with ZB, DL, and AB.

First, we identified relevant search terms, search strategies and information sources were conducted in July 2018, and the main search was carried out in November 2018. PubMed was used to systematically develop and test the search string before it was adapted and applied to other databases. In addition to PubMed, the academic databases Medline, Embase, Scopus, Web of Science, Cochrane, CINAHL and PsychInfo were included in the systematic search. The following basic search strings were used for all the databases:

((“emergency room” OR “emergency department” OR “emergency ward” OR “emergency patient” OR “emergency patients” OR “ED” OR “ER” OR “emergency medicine” OR “emergency medical service” OR “emergency medical services” OR “emergency departments” OR “emergency wards” OR “emergency unit” OR “emergency units” OR “emergency rooms” OR “emergency responders” OR “emergency responder”) AND (“IHM” OR “deaths” OR “mortalities” OR “death” OR “mortality”) AND (“boarding” OR “boarded” OR “overcrowded” OR “crowded” OR “crowding” OR “overcrowding” OR “access blocks” OR “access block” OR “lead-time” OR “admission delays” OR “delayed admissions” OR “patient admission” OR “patient admissions” OR “admission delay” OR “bed occupancy” OR “delayed transfer” OR “delayed admission” OR “bed management”)).

All terms were searched in the fields for “Abstract” and “Article Title” (alternatively in the field for “Topic”) and in the MeSH/Subject Headings/Thesaurus fields when available. All available publication years were included in the search. In order to retrieve the best possible result, to include grey references and avoid excluding pre-indexed materials. no additional filters or limitations were applied. The protocol was registered in PROSPERO, under number CRD42019119489 (https://www.crd.york.ac.uk/PROSPERO).

All references were uploaded to the reference management software EndNote and transferred to the reference and research management software F1000 (Computer software, London, UK) for deduplication, screening and data extraction. One additional reference was identified in additional cited and citing articles and was included in the systematic review in this process.

An updated search in PubMed to identify potential new papers published since the last search date was conducted before finalizing the manuscript. 36 references were retrieved in this search covering the publication period of 16/11/2018–29/05/2019. None of the 36 papers meet the study’s eligibility criteria.

A full search log including search dates, search strings, the results and notes about search technical specifications for each database, including the search update in PubMed, can be found in Appendix 1 (Literature Search).

### Selection process

Three reviewers who are qualified in emergency medicine and experts in ED operations (ZB, DL, AB) screened the titles and abstracts produced by the search independently beside the inclusion principles to identify relevant abstracts. They used F1000 Workspace (Computer software, London, UK), a reference management software program, to screen and select related articles. Articles with a title or abstract that did not meet at least one of the inclusion criteria were rejected. Complete reports were acquired for all titles that seemed to fit the inclusion criteria even if there existed any disagreement between reviewers or uncertainty. The three reviewers then read the full-text articles and selected those that met all inclusion criteria. Reviewers were not blinded to the titles of the journals, nor to the names and institutions of study authors. When necessary, we obtained additional information from study authors to resolve questions about eligibility. Data abstracted from the results and methods sections of every selected article included research setting, study population, research design, sample size, predictors, patient outcomes, and primary findings. When data in the initial study were not presented in a format that was valuable to reviewers, we asked the authors of included studies to provide relevant information for the review. Duplicate, overlapping, and companion studies were identified. Their data were combined into a single data collection Excel spreadsheet. We extracted data using a customized Excel spreadsheet including the following study characteristics: design, setting, population, sample size and primary objective.

Data on the following prognostic factors and outcomes were also extracted: boarding, ED LOS, crowding, type of mortality, outcomes (with measures of precision and significance), and adjustment for confounding factors (e.g., age, comorbidities, diagnosis, triage severity code). Missing information on prognostic factors or outcomes was requested from the authors. Three authors (ZB, DL, and AB) performed the data extraction separately. Discrepancies between reviewers were resolved by a fourth reviewer (MA). Finally, the study quality was rated and reported on the data collection form. Two authors (ZB and AB) performed quality assessments separately, and disagreements were determined by consensus in the presence of a third review author (MA). The study also reviewed the gray literature identified in the database search, including conference abstracts, but we excluded such studies when they did not have corresponding full-text articles published in scientific journals. Finally, we completed the PRISMA checklist (supportive information).

### Study quality assessment

We used the Newcastle-Ottawa Quality Assessment Scale (NOS) designed for nonrandomized trials to rate the quality of the selected studies [24]. The NOS consists of 4 items on “study selection”, 1 item on “comparability” and 3 items on “study outcome”. According to this scale, studies can be awarded one star for each of the 4 items on “selection” and for each of the 3 items on “outcome”, and a maximum of 2 stars for “comparability”. Two assessors (ZB, AB) performed the rating independently.

### Risk of bias

We used the modified RTI risk of bias tool for observational studies to evaluate the studies risk of bias [25,26]. The RTI item bank covers the most significant aspects involved in the NOS, and it allows for more thorough quality evaluation [27].

To evaluate the articles, the following domains were used: recruitment of participant strategy, inclusion criteria, exclusion criteria, comparison group selection, respect of the proposed protocol, study assessors blinding, measures of validity and reliability of the study, follow-up of length of the study, follow-up loss, missing important primary outcomes, assessment of problems in the study, research confounding factors and limitations.

### Data synthesis and analysis

We used a customized Excel spreadsheet to extract data on the following study characteristics: design, setting, population, sample size and main objective. This allowed us to create a comprehensive data summary of all included articles. The following data on in-hospital adverse outcomes were also extracted: LOS, mechanical ventilation, patient satisfaction, inpatient cost (IP cost), Methicillin-resistant Staphylococcus aureus (MRSA) infection, outcomes (with measures of precision and significance), and adjustment for confounding factors (e.g., age, comorbidities, diagnosis, triage severity code). Finally, the study quality was rated and reported on the data collection form. The odds ratio (OR) was chosen to measure the potential association between EDB and IHM. For a binary outcome variable, the measured effect usually expressed as a log of estimated OR. The weight is expressed as the inverse of the variance of log of estimated OR. We added for each study the effect estimate (e.g. odds ratio) with corresponding 95%- confidence interval.

## Results

### Overview

**Fig 1:**
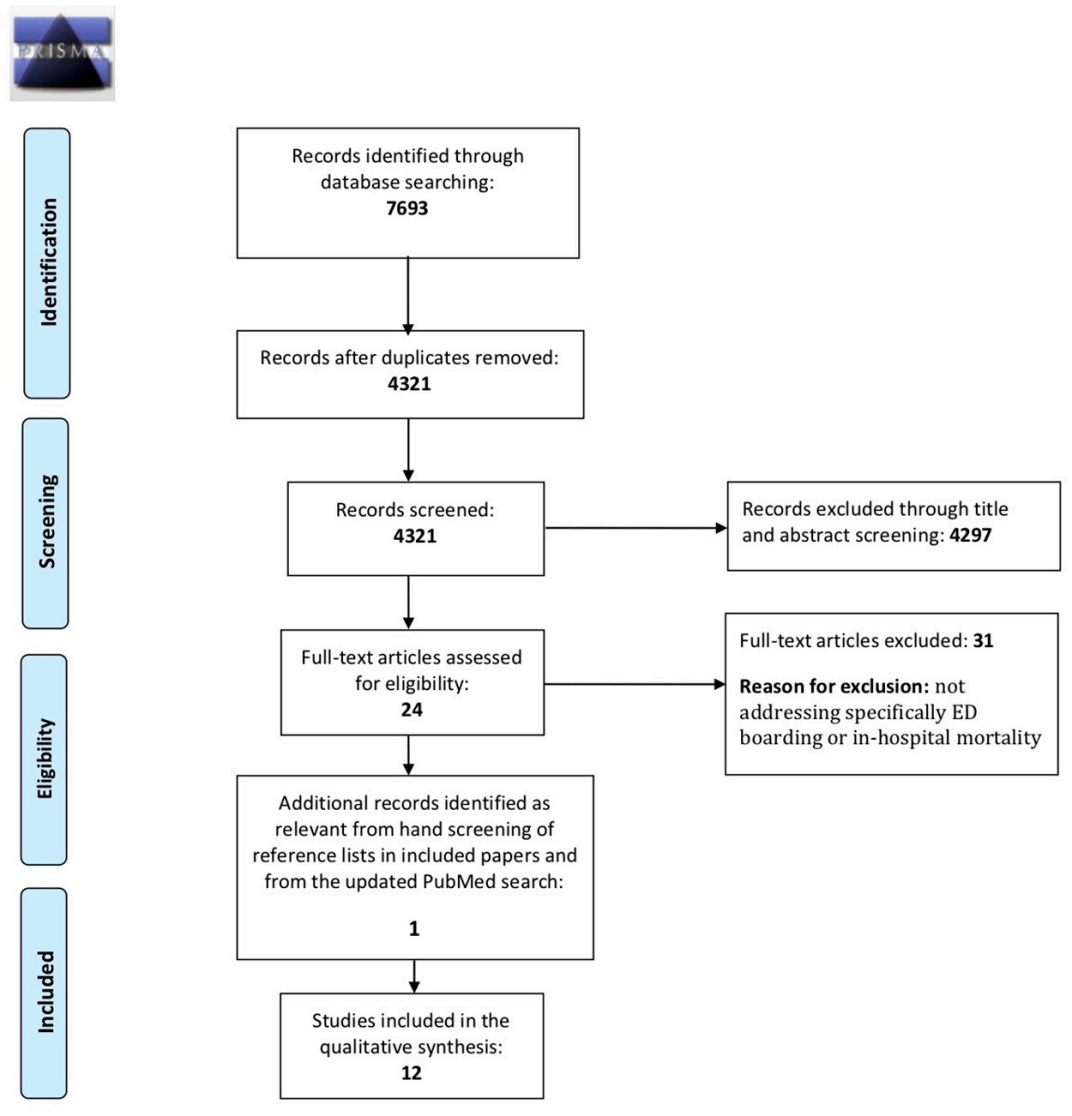
PRISMA flow diagram of study selection. *From*: Moher D, Liberati A, Tetzlaff J, Altman DG, The PRISMA Group (2009). *P*referred *R*eporting *I*terns for *S*ystematic Reviews and *M*eta-*A*nalyses: The PRISMA Statement. PLoS Med 6(7): e1000097. doi: 10.1371/journal.pmed.1000097 **For more information, visit** www.prisma-statement.org.

The initial database search identified a total of 4,321 studies. The selection process is showed in Fig 1. In the first screening step (titles and abstracts), 42 articles appeared to meet the eligibility criteria. Thus, a total of 42 studies underwent full-text review to assess for eligibility. Thirty one studies were excluded because they did not specifically address EDB or IHM. One study was added from the review of references. None of the studies from the updated literature search in PubMed in May 2019 were identified as relevant. Thus, 12 studies were eligible for descriptive analysis in our systematic review (Fig 1).

### Characteristics of included studies

The main characteristics of the included studies are summarized in Table 1. Twelve studies were selected [28–39]. Eight were retrospective cohort studies, three were cross-sectional, and one was a prospective observational study. The total number of patients included in these studies was 182,991.

**Table 1:** Characteristics of the selected studies.

**Authors /reference**	**Country**	**Title**	**Journal**	**ED setting**	**Study populations (admitted from the ED)**	**Study group (n)**	**ED boarding data type**	**Outcomes**
**Al-Qahtani et al**. [28] **2017**	Saudi Arabia	“The association of duration of boarding in the emergency room and the outcome of patients admitted to the intensive care unit”	“BMC Emergency Medicine”	1 hospital in Saudi Arabia	“ED* patients admitted to an ICU”	940	Quantitative (< 6 hrs)	“In-hospital mortality”
							Quantitative (6–24 hrs)	
							Quantitative (> 24 hrs)	
**Cha et al**. [29] **2015**	South Korea	“The impact of prolonged boarding of successfully resuscitated out-of-hospital cardiac arrest patients on survival-to-discharge rates.”	“Resuscitation”	Korean hospitals nationwide	“Successfully resuscitated OHCA *patients brought to the ED”	4,686	Quantitative cut-off (admission delay ≥ 6 hrs)	“In-hospital mortality”
**Chalfin et al**. [30] **2007**	USA	“Impact of delayed transfer of critically ill patients from the emergency department to the intensive care unit”	“Crit Care Med”	120 adult ICUs from 90 hospitals in the U.S.	“ED patients admitted to an ICU”	50,322	Quantitative (< 6 hrs)	“ICU and in-hospital mortality”
							Quantitative (≥ 6 hrs)	
**Hsieh et al**. [31] **2017**	Taiwan	“Impact of delayed admission to intensive care units on patients with acute respiratory failure”	“American Journal of Emergency Medicine”	1 hospital in Taiwan	“Adults with acute respiratory failure requiring ventilation support”	267	Quantitative (> 1 hr)	“In-hospital mortality”
							Quantitative (> 2 hrs)	
							Quantitative (> 4 hrs)	
**Gilligan et al**. [32] **2008**	Ireland	“The boarders in the emergency department (BED) study”	“Emerg Med Journal”	1 hospital in Ireland	“ED adults”	13,357	Continuous	“In-hospital mortality”
**Junhasavasdikul et al**. [33] **2012**	Thailand	“Association between admission delay and adverse outcome of emergency medical patients”	“Emerg Med Journal”	1 hospital in Thailand	“ED patients ≥ 15 years old”	381	Continuous	“In-hospital mortality”
**Singer et al**. [34] **2011**	USA	“The association between length of emergency department boarding and mortality”	“Society for Academic Emergency Medicine”	1 U.S. hospital	“ED patients admitted to inpatient wards”	41,256	Quantitative (< 2 hrs)	In-hospital “mortality”
							Quantitative (2–6 hrs)	
							Quantitative (6–12 hrs)	
							Quantitative (12–24 hrs)	
							Quantitative (> 24 hrs)	
**Augustin et al**. [35] **2017**	USA	“Impact of delayed admission to the intensive care unit from the emergency department upon sepsis outcomes and sepsis protocol compliance”	“Critical Care Research and Practice”	1 U.S. hospital	“ED patients admitted to an ICU with severe sepsis/septic shock”	287	Quantitative (< 6 hrs)	“In-hospital mortality”
**Lord et al**. [36] **2017**	USA	“Emergency department boarding and adverse hospitalization outcomes among patients admitted to a general medical service”	“American Journal of Emergency Medicine”	1 U.S. hospital	“ED patients admitted to a general medicine service”	31,219	Quantitative (≥ 6 hrs)	“Rapid response team activation, care escalation to ICU, in-hospital mortality”
							Quantitative (< 4 hrs)	
**Reznek et al**. [37] **2018**	USA	“Mortality associated with emergency department boarding exposure: Are there differences between patients admitted to ICU and non-ICU settings”	“Medical Care”	2 U.S. hospitals	“ED patients admitted to ICU and non-ICU wards”	39,781	Quantitative (≥ 4 hrs)	“In-hospital mortality”
							Continuous	
**Al-Khathaami et al**. [38] **2014**	Saudi Arabia	“The impact of ‘admit no bed’ and long boarding times in the emergency department on stroke outcome”	“Saudi Med J”	1 hospital in Saudi Arabia	“ED patients with stroke admitted to a medical ward”	300	Quantitative (0–0.75 hrs)	“In-hospital mortality and post-stroke complications”
							Quantitative (0.76–1.42 hrs)	
							Quantitative (1.43–2.97 hrs)	
							Quantitative (> 2.98 hrs)	
**Hong et al**. [39] **2008**	Taiwan	“The effects of prolonged ED stay on outcome in patients with necrotizing fasciitis”	“American Journal of Emergency Medicine”	1 hospital in Taiwan	“ED admitted patients with necrotizing fasciitis”	195	Quantitative (> 8 hrs)	“In-hospital mortality”
**Authors /reference**	**Test**	**ED boarding time**	**Data format (odds ratio, OR)**	**Data**	**P value**	**Adjustment factors**	**Comments/Conclusion**	
**Al-Qahtani et al**. [28] **2017**	Logistic regression analyses and stepwise multivariate linear regression analyses	< 6 hrs	OR (95% CI)	Reference	NA	“Age, sex, APACHE* score, mechanical ventilation, creatinine, platelets, INR”	"The study demonstrated an association between the duration of ED boarding of more than twenty-four hours with higher hospital mortality, duration of mechanical ventilation as well as increased total LOS".	
		6–24 hrs		1.54 (0.90, 2.70)	0.12			
		> 24 hrs		2.09 (1.22, 3.60)	0.007			
**Cha et al**. [29] **2015**	Logistic regression	Delay ≥ 6 hrs	OR (95% CI)	0.73 (0.62–0.86)	< 0.001	“Utstein factors and time intervals”	"Prolonged boarding of OHCA patients was associated with an increased mortality rate after adjustment. The influence was significant from 1 to 36 hours after ROSC"	
**Chalfin et al**. [30] **2007**	Stepwise backward logistic regression	Delay ≥ 6 hrs	OR (95% CI)	0.709 (0.561–0.895)	0.004	“Age, gender, APACHE* II score, GI* bleeding, coronary artery disease, drug overdose, polytrauma, intracerebral hemorrhage, neurologic disease, cardiovascular disease, CHF*, COPD*”	"Critically ill ED patients with a ≥ 6-hour delay in ICU transfer had increased hospital mortality".	
**Hsieh et al**. [31] **2017**	Stepwise backward regression	> 1 hr	OR (95% CI)	2.19 (1.04–4.64)	0.04	NA*	“Delayed ICU admission (> 1 hr) was a strong predictor of in-hospital crude mortality for patients w/ acute respiratory failure and ventilator support in the ED.”	
		> 2 hrs		Not available	NA			
		> 4 hrs		Not available	NA			
**Gilligan et al**. [32] **2008**	Logistic regression	Mean 16.1 hrs; Range (0–161 hrs)	OR (95% CI)	0.998 (0.983–0.992)	< 0.001	NA	“No conclusion drawn from duration of ED boarding and mortality. "Large numbers of boarders did not increase the likelihood of dying during admission for those patients who lived long enough to be admitted"	
**Junhasavasdikul et al**. [33] **2012**	Logistic regression	Lead Time (hrs)	OR (95% CI)	0.97 (0.93–1.01)	0.13	NA	“Shorter lead-time associated w/ increased mortality in univariate analysis, but association not found in multivariate analysis. "Might be explained by case selection and early treatment provided in ED"	
**Singer et al**. [34] **2011**	Logistic regression	< 2 hrs	OR (95% CI	Reference	< 0.001	“Age, sex, race, weekend, shift, and Elixhauser comorbidity variables”	“Emergency department boarding was associated with higher inpatient mortality rates and longer hospital length of stay in this hospital.”	
		2–6 hrs		0.91 (0.80–1.05)				
		6–12 hrs		1.24 (1.00–1.54)				
		12–24 hrs		1.43 (1.13–1.82)				
		> 24 hrs		1.23 (0.73–2.09)				
**Augustin et al**. [35] **2017**	Logistic regression	> 6 hrs	OR (95% CI)	1.226 (0.669–2.247)	0.51	"SOFA*, lactate, MAP*"	"There was no significant in-hospital mortality difference between critically ill septic patients admitted early to ICU versus those with delayed admission"	
**Lord et al**. [36] **2018**	Logistic regression	4 hrs	OR (95% CI)	0.82 (0.64–1.05)	NA	“Age, gender, insurance status, emergency severity index (ESI), Elixhauser comorbidity score, telemetry requirements”	"No significant association between boarding time and adverse hospital outcomes within 24 h of admission to general medicine but there was a significant association in regard to hospital outcomes that occurred at any time during the hospital stay"	
**Reznek et al**. [37] **2014**	Cox Proportional Hazards regression	patients who died in hospital	OR (95% CI)	1.2 fold risk (1.03–1.36)	NA	Not available	"Non-ICU patients who died in Hospital had higher risk of having experienced longer boarding times. However, we did not observe a difference among ICU patients"	
**Al-Khathaami et al**. [38] **2014**	Logistic regression	0–0.75 hrs	OR (95% CI)	…	…	“Age, sex, HTN*, diabetes, AF*, heart failure, previous stroke, hemorrhagic stroke, severity of stroke, onset to ED time, BT and ED wait time.”	"There was no association between BT and the primary outcome. Only a history of heart failure and previous stroke, in addition to the patient having a moderate to severe stroke were associated with adverse events"	
		0.76–1.42 hrs		0.7 (0.37–1.44)	0.36			
		1.43–2.97 hrs		1.2 (0.64–2.36)	0.51			
		> 2.98 hrs		0.5 (0.25–1.03)	0.06			
**Hong et al**. [39] **2008**	Stepwise backward logistic regression	Quantitative (> 8 hrs)	OR (95% CI)	3.4 (1.3–8.6)	0.012	NA	"We report an association between prolonged ED boarding stay and increased mortality in patients with necrotizing fasciitis".	

* APACHE score: acute physiology and chronic health evaluation score, SOFA: sequential organ failure assessment/sepsis, MAP: mean arterial pressure, TDD: time to decision to admit, CHF: congestive heart failure, COPD: chronic obstructive pulmonary disease, GI: gastro-intestinal, LOS: length of stay, BT: boarding time, OHCA: out-of-hospital cardiac arrest, ICU: intensive care unit, HTN: hypertension, AF: atrial fibrillation., ED: emergency department, OR: operating room, MICU: medical intensive care unit.

Five studies were performed in the USA [30,34–37]. Two studies were realized in Taiwan [31,39], one in South Korea [29], one in Thailand [33], two in Saudi Arabia [28,38], and one in Ireland [32].

A total of five studies investigated exclusively patients admitted to intensive care units (ICU). Two of these studies included ICU admissions for any type of condition [28,30] and three studies included ICU admissions for specific medical conditions: one study investigated cardiac arrest of out-of-hospital patients who boarded in the ED before being admitted [29], and the other two studies included patients with acute respiratory failure [31] and sepsis/septic shock patients [35], respectively.

A total of seven studies included non-ICU patients. Of these, three studies included all ED patients admitted to an inpatient bed [32,34,36], two studies included ED patients admitted with specific conditions such as stroke [38] and necrotizing fasciitis [39], and two studies included subgroup analyses for ED patients admitted to ICU and non-ICU inpatient wards [33,37].

### Outcomes

**Fig 2:**
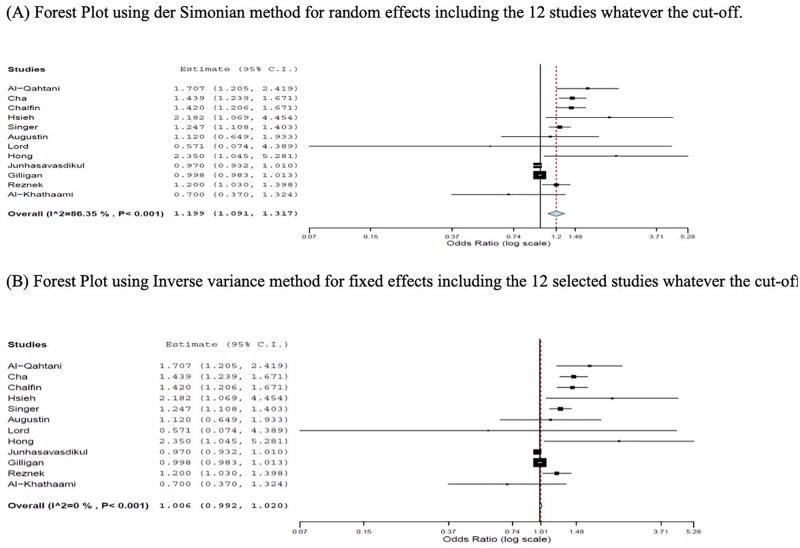
Forest plots for the estimation of the size effect. (A) Forest Plot using der Simonian method for random effects including the 12 studies whatever the cut-off. (B) Forest Plot using Inverse variance method for fixed effects including the 12 selected studies whatever the cut-off.

Among the twelve selected studies, six studies showed a significant association between ED boarding and IHM [28–31,34,39], five studies showed no association [32,33,35,36,38], and one study [37] that investigated patients admitted to ICU and non-ICU wards showed conflicting results, with a positive association for non-ICU patients but none for ICU patients. The results are summarized in Table 2. Odds ratio with corresponding 95%- confidence intervals are shown in Table 3. A tendency toward an association between EDB and IHM using the pool random effect is shown in Fig 2. But, different cut-offs are pooled which may not be methodologically appropriate. Analysis of the tendency study by study found an association in seven studies [28–31,34,37,39]. We did not perform a meta-analysis which is the most appropriate method to find the overall estimated effect size and to evaluate the heterogeneity between different studies that can confirm our hypothesis. Because few selected studies have the same cut-offs, total of four studies do not provide the meaningful and valid picture.

**Table 2:** Results summary: Association between Emergency Department (ED) boarding and In-hospital mortality.

Authors /reference	Title	Association of ED boarding with in-hospital mortality Yes/ No
**Al-Qahtani et al**. [28]	“The association of duration of boarding in the emergency room and the outcome of patients admitted to the intensive care unit”	**Yes**
**Cha et al**. [29]	“The impact of prolonged boarding of successfully resuscitated out-of-hospital cardiac arrest patients on survival-to-discharge rates.”	**Yes**
**Chalfin et al**. [30]	“Impact of delayed transfer of critically ill patients from the emergency department to the intensive care unit”	**Yes**
**Hsieh et al**. [31]	“Impact of delayed admission to intensive care units on patients with acute respiratory failure”	**Yes**
**Gilligan et al**. [32]	“The boarders in the emergency department (BED) study”	**No**
**Junhasavasdikul et al**. [33]	“Association between admission delay and adverse outcome of emergency medical patients”	**No**
**Singer et al**. [34]	“The association between length of emergency department boarding and mortality”	**Yes**
**Augustin et al**. [35]	“Impact of delayed admission to the intensive care unit from the emergency department upon sepsis outcomes and sepsis protocol compliance”	**No**
**Lord et al**. [36]	“Emergency department boarding and adverse hospitalization outcomes among patients admitted to a general medical service “	**No**
**Reznek et al**. [37]	“Mortality associated with emergency department boarding exposure: Are there differences between patients admitted to ICU and non-ICU settings”	**Yes**: for non-ICU-admitted patients **No**: for ICU-admitted patients
**Al-Khathaami et al**. [38]	“The impact of “admit no bed” and long boarding times in the emergency department on stroke outcome”	**No**
**Hong et al**. [39]	“The effects of prolonged ED stay on outcome in patients with necrotizing fasciitis”	**Yes**

**Table 3:** Crude data collected in the control group non-exposed to boarding time and experimental group exposed to the boarding time over the cut-off reported in the selected studies.

Study	Boarding time cut-off (hours)	Study group (n)	Exposed (research group) >Boarding time			Not exposed (control group) <Boarding time			Odds ratio	CI (95%)	Comments
			Total	Dead	Alive	Total	Dead	Alive			
Al-Qahtani et al. [28]	6	940	713	236	477	227	51	176	1.707	1.205–2.419	
Cha et al. [29]	6	4,686	1267	974	293	3419	2386	1033	1.439	1.239–1.671	
Chalfin et al. [30]	6	50,322	1036	180	856	49286	6358	42928	1.420	1.206–1.671	
Hsieh et al. [31]	1	267	200	60	140	67	11	56	2.182	1.069–4.454	
Gilligan et al. [32]	Continuous Mean 16.1 Range (0–161)	13,357	NA	NA	NA	NA	NA	NA	0.998	0.983–1.013	Calculated upper value of CI
Junhasavasdikul et al. [33]	Continuous Lead Time	381	NA	NA	NA	NA	NA	NA	0.970	0.932–1.01	
Singer et al. [34]	2	41,256	20729	642	20087	20527	513	20014	1.247	1.108–1.403	
Augustin et al. [35]	6	287	150	37	113	137	31	106	1.120	0.649–1.933	
Lord et al. [36]	4	31,219	3978	1	3977	27,241	12	27,229	0.571	0.074–4.389	
Reznek et al. [37]	Continuous	39,781	23	NA	NA	NA	NA	NA	1.2	1.03–1.398	Calculated upper value of CI
Al-Khathaami et al. [38]	0.75	300	213	NA	NA	75	NA	NA	0.7	0.37–1.324	Calculated upper value of CI
Hong et al. [39]	8	195	70	15	55	125	13	112	2.350	1.045–5.281	

#### Patients admitted to an ICU

Five studies included only ED patients who were admitted to an ICU [28–31,35] and two other studies included subgroups of ICU patients [33,37]. Thus, a total of seven studies included patients admitted to an ICU. Among the seven studies and subgroups, four found a positive association between ED boarding and IHM [28–31] and three studies did not find any association [33,35,37].

Among the four studies that found a positive association, the study by Al-Qahtani et al. [28] showed a statistically significant association between EDB and IHM, as well as an association of EDB with increased ICU mortality and ICU LOS. A delayed admission was an independent risk factor for hospital and ICU mortality (difference between EDB less than six hours vs. more than twenty-four hours). Cha et al. [29] study investigated the effect of EDB on survival in patients with successfully resuscitated out-of-hospital cardiac arrest. After adjustment, it was found that prolonged boarding increases IHM. This impact was significant from 1 to 36 hours after ROSC [29]. Likewise, Chalfin et al. [30] showed an association between EDB and higher IHM, ICU mortality and hospital LOS. Critically ill ED patients with a delay over six hours in the admission to ICU had higher ICU and IHM and increased hospital LOS. Hsieh et al. [31] included ED patients with acute respiratory failure who were admitted to an ICU and showed that IHM was correlated with ED boarding time. This study demonstrated that a delay of more than one hour in the ICU admission is a factor of mortality, prolonged mechanical ventilation, and an increased ICU stay.

In contrast to the above studies, three studies showed no association between ED boarding and IHM [33,35,37]. One retrospective cohort study including ED patients with sepsis admitted to an ICU found no significant difference in clinical outcomes between patients with delayed ICU admission and patients with early ICU admission, moreover there was no impact on sepsis protocol compliance [35]. Two studies evaluated ED patients admitted to both ICU and non-ICU inpatient wards [33,37]. In the study by Junhasavasdikul et al. [33], EDB was not a risk factor of IHM. In the other study, Reznek et al. [37] observed no difference in IHM among ICU patients who experienced EDB.

#### Non-ICU patients

A total of seven studies included non-ICU patients; five included exclusively non-ICU patients [32,34,36,38,39] and two studies included subgroups of non-ICU patients [33,37]. Among these seven studies and subgroups, three studies showed a positive association between EDB and IHM [34,37,39] whereas four showed no association [32,33,36,38].

Among the studies that showed a positive correlation, Singer et al. [34], showed that EDB was associated with increased hospital LOS and IHM. In patients boarding less than 2 hours, mortality was lower than those who stayed 12 hours (2.5% vs 4.5%) [34]. In another study, Reznek et al. [37] compared the effect of boarding on patients who were admitted to ICU to patients who were admitted to non-ICU wards. The non-ICU patients who survived had shorter boarding in the ED than patients who died during hospitalization [37]. Likewise, the study by Hong et al. [39] demonstrated that prolonged EDB was dependent on increased mortality for ED patients with a specific diagnosis of necrotizing fasciitis (OR 3.4; 95% CI 1.3–8.6); hypotension was also dependent on higher in-hospital mortality (OR 32.9; 95% CI 6.9–156.0).

In contrast to the above studies, four studies demonstrated no association between EDB and IHM. Gilligan et al. [32] found no association between EDB and IHM. In the study by Junhasavasdikul et al. [33], EDB was independent of IHM. The third study focused on the relationship between EDB and in-hospital outcomes for the specific population of ED patients admitted only to general medicine wards [36]. The aim was to investigate the association between EDB and outcomes of hospitalization and adverse consequences that may portray harm of lesser severity than mortality. Since there were few existing studies on general medicine admissions; the authors concluded that within the first 24 hours of admission to a hospital to a medical ward, adverse events (rapid response team activation, care escalations, unanticipated in-hospital mortality) were rare and not associated with EDB [36]. There was no relation between EDB and adverse hospital outcomes within 24 hours of general medicine ward admission. Al-Khataami et al. [38] included ED patients with a specific diagnosis of stroke and showed that there was no association between EDB and IHM and/or any post stroke complications, nor for secondary outcomes (severe disability, pneumonia, urinary tract infection, neurological deterioration); only stroke, heart failure, and previous stroke predicted poor outcomes.

### Quality of the studies

NOS was used in all the studies for evaluation. Nine studies attained moderate ratings [28,29,31–33,35,36,38,39] and three studies achieved high scores overall [30,34,37]. The quality assessment is reported in Table 4.

**Table 4:** Quality assessment of the studies (Newcastle-Ottawa quality assessment scale).

Authors/ reference	Selection				Comparability of cohorts	Outcome			Total Score T
	Representativeness of the exposed cohort	Selection of the nonexposed cohort	Ascertainment of exposure	Outcome of interest not present at study start		Assessment	Length of follow-up	Adequacy of follow-up of cohorts	
**Al-Qahtani et al**. [28]	*		*	*	*	*			6
**Cha et al**. [29]	*	*			*	*		*	5
**Chalfin et al**. [30]	*	*	*	*	*	*	*	*	8
**Hsieh et al**. [31]			*	*	*	*	*	*	6
**Gilligan et al**. [32]	*		*		*	*	*		5
**Junhasavasdikul et al**. [33]	*		*	*	*	*			5
**Singer et al**. [34]	*	*	*	*		*	*	*	7
**Augustin et al**. [35]		*		*	*	*	*		5
**Lord et al**. [36]		*	*	*	*	*		*	6
**Reznek et al**. [37]	*	*	*	*	*	*	*	*	8
**Al-Khathaami et al**. [38]	*		*	*	*	*			5
**Hong et al**. [39]	*	*	*		*	*		*	6

### Evaluation of risk of bias

Overall, most articles had credible results. The majority of studies (66%, n = 8) did not adequately take into account all the confounding variables in the design and analysis. Due to the design of most studies (retrospective cohort, cross-sectional), six entries from the risk of bias tool were inapplicable (Table 5).

**Table 5:** Evaluation of risk of bias.

Authors/ reference	1. Inclusion /exclusion varies between groups	2. Recruitment varies between groups	3. Sample size sufficiently large	4. Description of the intervention or exposure	5. Outcome assessor blinded	6. Valid and reliable measures	7. Differential follow-up	8. Impact of highloss to follow-up	9. Missing outcomes	10. Missing harm or adverse event data	11. Results believable	12. Balance of allocation between groups	13. Confounding addressed appropriately
**Al-Qahtani et al**. [28]	**+**	**+**	?	?	?	+	?	?	+	?	+	?	Part
**Cha et al**. [29]	**+**	?	?	?	?	+	+	?	+	?	+	?	Part
**Chalfin et al**. [30]	**+**	+	+	?	?	+	?	?	+	?	+	?	Part
**Hsieh et al**. [31]	**+**	+	?	?	?	?	?	?	+	?	+	?	Part
**Gilligan et al**. [32]	**+**	?	?	?	?	?	?	?	+	?	+	?	-
**Junhasavasdikul et al**. [33]	**+**	?	?	?	?	+	?	?	+	?	+	?	-
**Singer et al**. [34]	**+**	?	?	?	?	+	?	?	+	?	+	?	+
**Augustin et al**. [35]	**+**	?	?	?	?	+	?	?	+	?	+	?	Part
**Lord et al**. [36]	**+**	**+**	?	?	?	+	?	?	+	?	+	?	+
**Reznek et al**. [37]	**+**	+	?	+	?	+	+	?	+	?	+	?	Part
**Al-Khathaami et al**. [38]	**+**	?	-	?	?	-	?	?	**+**	?	**+**	?	Part
**Hong et al**. [39]	**+**	**+**	**+**	?	?	?	+	?	**+**	?	**+**	?	Part

+ Study met criteria,—Study did not meet criteria, ? Not applicable, Part: partially met criteria

## Discussion

Our systematic review did not find strong evidence of increased IHM related to EDB but it seems that there is a tendency toward an association between EDB and IHM as shown in the forest plots using the pool random effect (Fig 2). However, using pooled estimates may not be methodologically appropriate. Nevertheless, this tendency was also observed with the analysis study by study (Table 3). More studies are needed to allow a complete meta-analysis which may confirm this tendency.

EDB is an important public health issue that has been associated with adverse patient outcomes, such as delays in antibiotic administration [6], delays in pain medication administration [8,40,41], lower patient satisfaction [41], prolonged times to disposition among patients with acute asthma [42], and higher complication rates for cardiovascular events [43]. Furthermore, ED patients who are waiting for ICU beds seem to be at higher risk of IHM and adverse outcomes. The literature targeting this area of research has grown, but surprisingly only just above half of the studies we selected showed an association between ED boarding and IHM [28–31,34,39]. Unfortunately, it was not possible to conduct a meta-analysis because of variation in the EDB time cut-offs.

It was shown that critically sick patients have better results when treated in ICUs [44–46]. Critically sick patients are expected to survive when critical care starts quickly [44–48], and delayed admission is more likely to be linked with increased risk of mortality [20,49]. In our systematic review, more than half of the studies involving ICU patients and showed an association between EDB and IHM [28–31]. Many factors may have contributed to this association. One possible explanation is that the healthcare providers in the ED are not all trained in critical care medicine and managing critically ill patients, the ED is not appropriate [28]. Another potential reason is that the quality of care is not optimal with delayed time-sensitive treatments and increased medical errors [29]. Moreover, the busy, fast-paced nature of the ED would not allow ED physicians and nurses to propose the focused one-on-one care that a critically sick patient may need and will get in the ICU. The lesser expertise in critical care among ED physicians and nurses compared to ICU teams [30] is an important factor associated with the lack of close monitoring compared to ICU settings and the nurse-to-patient ratio [31]. These findings were concordant with some studies that demonstrated that when ICU admission is delayed, there is an increase in mortality [20,50,49]. Delayed admission increases the risk of requiring mechanical ventilation, renal replacement therapy, and the need of resources [51]. Various studies have revealed that patients who are critically sick have better results when treated in ICUs with continuous and close involvement by critical care physicians [44–46] and showed improved results when nurse-to-patient ratios in the intensive care units are accurately kept [52]. Another potential factor that could impact the boarders’ patients’ outcomes is whether the team managing critical patients in the ED is an ED team or an ICU team posted in the ED. In many countries, particularly those where the specialty of emergency medicine exists, emergency physicians are trained to manage those patients during the ED stay. Difficulties are observed when patients are staying an abnormal longer of time than usually recommended and need specific ICU care which is not the role of the ED.

Three studies investigating ED patients admitted to ICUs found no association between EDB and IHM [33,35,37]. There are various possible explanations for these negative results. The early treatment provided by the ED staff for critical patients may have attenuated the impact of EDB, suggesting that the adequate time to treatment could be the factor that defined mortality as compared to the boarding time [33]. The standardization of care for ED patients with sepsis, thanks to the widespread use of sepsis protocols may have contributed to reducing deaths among septic ED patients [35]. The implementations of countermeasures, such as prioritizing the perceived sickest ICU patients for available beds may have mitigated the potential mortality effects of boarding for ED patients who were admitted to ICUs [37]. These results confirmed previous studies showing no effect on survival in critically sick patients whatever the ICU admission delay [53,54].

Most of the studies that investigated the association between EDB and IHM were retrospective, and we can only analyze the relationship between predictors and outcomes. Risk factors and causality would be ideally assessed by clinical trials comparing prolonged ED boarding and immediate transfer. Because of ethical issues associated with such studies, ‘pre-post’ design studies evaluating interventions to reduce EDB and their effects on in-hospital outcomes would be more suitable. In two studies [55,56] the authors included ICU patients and used an interesting ‘pre-post design’ that investigated the effects of reducing EDB on IHM and adverse outcomes. The two studies had similar objectives, aiming to reduce EDB for patients waiting for ICU beds using two different tools; one study added a doctor in charge of facilitating the admission of ED patients, and the other study changed the admission policy. Both studies showed positive effect on the management of ED critically ill patients who are admitted to ICUs with a streamlined admission process. These findings were in concordance with those of other studies (49,50,57,58). In ED patients with septic shock, mortality was significantly reduced after early goal-directed therapy was quickly implemented as soon as the diagnosis was made [49]. Increased survival was also observed when trauma victims were quickly transferred to trauma centers [50]. Similarly, ischemic cerebrovascular events [57] and acute myocardial infarction patients have better survival when they are admitted to the right ward unit [58].

The second category of studies included non-ICU patients. Similar to the findings for ICU patients, the results regarding the association between EDB and IHM were conflicting among patients who were presumably less severely ill. Several potential contributing factors were identified in the studies that demonstrated a positive association between EDB and IHM [34,37,39]: ED crowding resulting in less time given to ED boarders, delays in testing and therapeutic interventions, and increase in severity of condition with prolonged ED boarding [34]. The absence of a clear model for admission of non-ICU patients and inefficient mixed-responsibility models for this category of ED patients were also potential explanations [37]. These findings confirm those of other studies showing that boarding is a risk factor of negative in-hospital outcomes [21,22]. However, four studies showed no association between EDB and IHM [32,33,36,38]; this may be explained by the potential benefits of various treatments initiated in ED [33] and the lower risk of harm to patients admitted to general medicine with less severe conditions [36,38]. ED crowding is defined as “the situation in which ED function is impeded primarily because of the excessive number of patients waiting to be seen, undergoing assessment and treatment, or waiting for departure comparing to the physical or staffing capacity of the ED” [59]. There is a lack of full data in the selected studies on ED crowding during the time of ED boarding and its potential effects on patients’ outcomes. This is mainly due to the retrospective nature of the majority of studies selected during the screening of the literature. Similarly, data regarding to other factors influencing ED crowding are also missing, e.g. the total volume of patients in ED during the times of long ED boarding, the nature of severity of patients treated in the ED, the ratio of ED doctors/patients, the existence of protocols for escalation in case of excessive ED crowding or ED boarding, the total capacity of the hospital available beds. Nevertheless, even though the impact of ED crowding has been found to have negative impact on patients’ outcomes [2–8], it does not seem to have systematic negative impact of ED boarding in this systematic review.

Our systematic review showed striking discrepancies between studies investigating the association between ED boarding and IHM. There was no consensus on an association when considering all study settings or admission units (ICU, non-ICU, inpatient ward, general medicine). There was a similar lack of consensus whether the entire ED population was considered or only specific target subgroups with specific conditions were included. Furthermore, for those studies that mentioned the team managing ED patients there was no clear relationship between ED boarding and IHM whether they were being managed by the ED team or the ED-based inpatient teams (medical ward or ICU), in case of crisis in the ED. However, this fact seems to be more related to the efficiency of ED-based ICU or medical teams and its impact on patients’ outcomes [60], rather than the impact of ED boarding itself. In our estimation, these findings reflect the results of the overall peer-reviewed literature.

More significantly, all the authors, even those who did not find a positive association between ED boarding and IHM, emphasized the importance of the harms resulting from EDB. These authors universally urged caution against oversimplification of the interpretation of their study results and refuted any suggestion that the ED can be a safe place for boarding patients.

### Limitations

Our systematic review has several limitations. First, there was significant heterogeneity among studies regarding the cut-off time to define EDB. This is due to a lack of a unique cut-off time to define EDB or prolonged ED LOS. Prolonged ED visits have variably been defined as over 4 hours in the United Kingdom, over 6 hours in Canada/US, and over 8 hours in Australia [61–63]. It was not possible to perform a full meta-analysis because of the low number of studies with the same cut-off which may allow to confirm the tendency toward an association between EDB and IHM. Second, the majority of studies included were observational and retrospective, which would not allow the authors to determine precisely whether EDB was due to a lack of availability of hospital beds or other barriers to treatment or assessment. Furthermore, due to the observational nature of the studies, some data could not be obtained (e.g., ED capacity, number and type of physicians, nursing staff, volume of critically ill patients at the time of admission and other factors such as co-morbidities). These factors may have caused a change in the overall results. Additionally, in some studies a subtle evolution of illness in the ED may occur and could have influenced the timing and type of admission (ICU, non-ICU), thus influencing the outcomes. There was heterogeneity among the selected studies in the ED population chosen with regard to the severity of disease, the type of admission (ICU, non-ICU) and the study settings. These differences may have induced selection bias. Nevertheless, we did analyze the results globally and by subgroups in order to identify group-specific results and details. Lastly, there was significant heterogeneity in the length of patients’ follow-up (from 24 hours to several months), so we were unable to assess long-term outcomes or contributing factors. Data on long-term outcomes would give us a better understanding of the long-term consequences of ED boarding.

## Conclusions

Our systematic review did not show strong evidence that EDB increases IHM but it shows a tendency toward an association between EDB and IHM. Just above half of the selected studies (58%) found an association between EDB and IHM. There is widespread focus on EDB because of its relationship with ED crowding and increased LOS, which can negatively impact the quality and safety of care. EDB is a significant source of patient dissatisfaction and it is associated with medical errors and adverse events including death. The heterogeneity and risk of bias combined with the low number of studies with the same cut-off could not allow to perform a meta-analysis. Prospective international multicenter studies are needed to clarify our findings. Nevertheless, our systematic review highlights a clear and shared message delivered by all authors, which is that EDB may cause harm to patients waiting for an in-hospital bed. The authors emphasize the absolute necessity to implement efficient interventions to minimize EDB.

## Supporting information

S1 Table Characteristics of the selected studies. (DOCX)

S2 Table Results summary: Association between Emergency Department (ED) boarding and in-hospital mortality. (DOCX)

S3 Table Crude data collected in the control group non-exposed to boarding time and experimental group exposed to the boarding time over the cut-off reported in the selected studies. (DOCX)

S4 Table Quality assessment of the studies (Newcastle-Ottawa quality assessment scale). (DOCX)

S5 Table Evaluation of risk of bias. (DOCX)

S6 Table PRISMA checklist. (DOCX)

S1 Appendix Literature search. (DOCX)
