# Supplementary material for: Association between boarding in the emergency department and in-hospital mortality: A systematic review
Source: PLoS One. 2020 Apr 15;15(4):e0231253. doi: 10.1371/journal.pone.0231253 (PMC7159217; doi:10.1371/journal.pone.0231253)
Supplement: S1 Table — (DOCX) [file pone.0231253.s001.docx]

**Table 1. Characteristics of the Selected Studies.**

| **Authors /reference** | **Country** | **Title** | **Journal** | **ED setting** | **Study populations (admitted from the ED)** | **Study group**  **(n)** | **ED boarding data type** | **Outcomes** |
| --- | --- | --- | --- | --- | --- | --- | --- | --- |
| **Al-Qahtani et al. [28]** | Saudi Arabia | The association of duration of boarding in the emergency room and the outcome of patients admitted to the intensive care unit | BMC Emergency Medicine | 1 hospital in Saudi Arabia | ED* patients admitted to an ICU | 940 | Quantitative (< 6 hrs) | In-hospital mortality |
|  |  |  |  |  |  |  | Quantitative (6-24 hrs) |  |
|  |  |  |  |  |  |  | Quantitative (> 24 hrs) |  |
| **Cha et al. [29]** | South Korea | The impact of prolonged boarding of successfully resuscitated out-of-hospital cardiac arrest patients on survival-to-discharge rates. | Resuscitation | Korean hospitals nationwide | Successfully resuscitated OHCA *patients brought to the ED | 4,686 | Quantitative cut-off (admission delay > 6 hrs) | In-hospital mortality |
| **Chalfin et al. [30]** | USA | Impact of delayed transfer of critically ill patients from the emergency department to the intensive care unit | Crit Care Med | 120 adult ICUs from 90 hospitals in the U.S. | ED patients admitted to an ICU | 50,322 | Quantitative (< 6 hrs) | ICU and in-hospital mortality |
|  |  |  |  |  |  |  | Quantitative (> 6 hrs) |  |
| **Hsieh et al. [31]** | Taiwan | Impact of delayed admission to intensive care units on patients with acute respiratory failure | American Journal of Emergency Medicine | 1 hospital in Taiwan | Adults with acute respiratory failure requiring ventilation support | 267 | Quantitative (> 1 hr) | In-hospital mortality |
|  |  |  |  |  |  |  | Quantitative (> 2 hrs) |  |
|  |  |  |  |  |  |  | Quantitative (> 4 hrs) |  |
| **Gilligan et al. [32]** | Ireland | The boarders in the emergency department (BED) study | Emerg Med Journal | 1 hospital in Ireland | ED adults | 13,357 | Continuous | In-hospital mortality |
| **Junhasavasdikul et al. [33]** | Thailand | Association between admission delay and adverse outcome of emergency medical patients | Emerg Med Journal | 1 hospital in Thailand | ED patients > 15 years old | 381 | Continuous | In-hospital mortality |
| **Singer et al. [34]** | USA | The association between length of emergency department boarding and mortality | Society for Academic Emergency Medicine | 1 U.S. hospital | ED patients admitted to inpatient wards | 41,256 | Quantitative (< 2 hrs) | In-hospital mortality |
|  |  |  |  |  |  |  | Quantitative (2-6 hrs) |  |
|  |  |  |  |  |  |  | Quantitative (6-12 hrs) |  |
|  |  |  |  |  |  |  | Quantitative (12-24 hrs) |  |
|  |  |  |  |  |  |  | Quantitative (> 24 hrs) |  |
| **Augustin et al. [35]** | USA | Impact of delayed admission to the intensive care unit from the emergency department upon sepsis outcomes and sepsis protocol compliance | Critical Care Research and Practice | 1 U.S. hospital | ED patients admitted to an ICU with severe sepsis/septic shock | 287 | Quantitative (< 6 hrs) | In-hospital mortality |
| **Lord et al. [36]** | USA | Emergency department boarding and adverse hospitalization outcomes among patients admitted to a general medical service | American Journal of Emergency Medicine | 1 U.S. hospital | ED patients admitted to a general medicine service | 31,219 | Quantitative (> 6 hrs) | Rapid response team activation, care escalation to ICU, in-hospital mortality |
|  |  |  |  |  |  |  | Quantitative (< 4 hrs) |  |
| **Reznek et al. [37]** | USA | Mortality associated with emergency department boarding exposure: Are there differences between patients admitted to ICU and non-ICU settings | Medical Care | 2 U.S. hospitals | ED patients admitted to ICU and non-ICU wards | 39,781 | Quantitative (> 4 hrs) | In-hospital mortality |
|  |  |  |  |  |  |  | Continuous |  |
| **Al-Khathaami et al. [38]** | Saudi Arabia | The impact of “admit no bed” and long boarding times in the emergency department on stroke outcome | Saudi Med J | 1 hospital in Saudi Arabia | ED patients with stroke admitted to a medical ward | 300 | Quantitative  (0 - 0.75 hrs) | In-hospital mortality and post-stroke complications |
|  |  |  |  |  |  |  | Quantitative  (0.76 - 1.42 hrs) |  |
|  |  |  |  |  |  |  | Quantitative  (1.43 - 2.97 hrs) |  |
|  |  |  |  |  |  |  | Quantitative  (> 2.98 hrs) |  |
| **Hong et al. [39]** | Taiwan | The effects of prolonged ED stay on outcome in patients with necrotizing fasciitis | American Journal of Emergency Medicine | 1 hospital in Taiwan | ED admitted patients with necrotizing fasciitis | 195 | Quantitative (> 8 hrs) | In-hospital mortality |

**Table 1 (Continued). Characteristics of the Selected Studies.**

| **Authors /reference** | **Test** | **ED boarding time** | **Data format (odds ratio, OR)** | **Data** | **P value** | **Adjustment factors** | **Comments/Conclusion** |
| --- | --- | --- | --- | --- | --- | --- | --- |
| **Al-Qahtani et al. [28]** | Logistic regression analyses and stepwise multivariate linear regression analyses | < 6 hrs | OR (95% CI) | Reference | NA | Age, sex, APACHE* score, mechanical ventilation, creatinine, platelets, INR | "The study demonstrated an association between the duration of ED boarding of more than twenty-four hours with higher hospital mortality, duration of mechanical ventilation as well as increased total LOS". |
|  |  | 6-24 hrs |  | 1.54 (0.90, 2.70) | 0.12 |  |  |
|  |  | > 24 hrs |  | 2.09 (1.22, 3.60) | 0.007 |  |  |
| **Cha et al. [29]** | Logistic regression | Delay > 6 hrs | OR (95% CI) | 0.73 (0.62-0.86) | < 0.001 | Utstein factors and time intervals | "Prolonged boarding of OHCA patients was associated with an increased mortality rate after adjustment. The influence was significant from 1 to 36 hours after ROSC" |
| **Chalfin et al. [30]** | Stepwise backward logistic regression | Delay > 6 hrs | OR (95% CI) | 0.709 (0.561-0.895) | 0.004 | Age, gender, APACHE* II score, GI* bleeding, coronary artery disease, drug overdose, polytrauma, intracerebral hemorrhage, neurologic disease, cardiovascular disease, CHF*, COPD* | "Critically ill ED patients with a > 6-hour delay in ICU transfer had increased hospital mortality". |
| **Hsieh et al. [31]** | Stepwise backward regression | > 1 hr | OR (95% CI) | 2.19 (1.04-4.64) | 0.04 | NA* | Delayed ICU admission (> 1 hr) was a strong predictor of in-hospital crude mortality for patients w/ acute respiratory failure and ventilator support in the ED. |
|  |  | > 2 hrs |  | Not available | NA |  |  |
|  |  | > 4 hrs |  | Not available | NA |  |  |
| **Gilligan et al. [32]** | Logistic regression | Mean 16.1 hrs; Range (0-161 hrs) | OR (95% CI) | 0.998 (0.983-0.992) | < 0.001 | NA | No conclusion drawn from duration of ED boarding and mortality. "Large numbers of boarders did not increase the likelihood of dying during admission for those patients who lived long enough to be admitted". |
| **Junhasavasdikul et al. [33]** | Logistic regression | Lead Time (hrs) | OR (95% CI) | 0.97 (0.93-1.01) | 0.13 | NA | Shorter lead-time associated w/ increased mortality in univariate analysis, but association not found in multivariate analysis. "Might be explained by case selection and early treatment provided in ED". |
| **Singer et al. [34]** | Logistic regression | < 2 hrs | OR (95% CI | Reference | < 0.001 | Age, sex, race, weekend, shift, and Elixhauser comorbidity variables | Emergency department boarding was associated with higher inpatient mortality rates and longer hospital length of stay in this hospital. |
|  |  | 2-6 hrs |  | 0.91 (0.80-1.05) |  |  |  |
|  |  | 6-12 hrs |  | 1.24 (1.00-1.54) |  |  |  |
|  |  | 12-24 hrs |  | 1.43 (1.13-1.82) |  |  |  |
|  |  | > 24 hrs |  | 1.23 (0.73-2.09) |  |  |  |
| **Augustin et al. [35]** | Logistic regression | > 6 hrs | OR (95% CI) | 1.226 (0.669-2.247) | 0.51 | "SOFA*, lactate, MAP*" | "There was no significant in-hospital mortality difference between critically ill septic patients admitted early to ICU versus those with delayed admission". |
| **Lord et al. [36]** | Logistic regression | 4 hrs | OR (95% CI) | 0.82 (0.64-1.05) | NA | Age, gender, insurance status, emergency severity index (ESI), Elixhauser comorbidity score, telemetry requirements | "No significant association between boarding time and adverse hospital outcomes within 24 h of admission to general medicine but there was a significant association in regard to hospital outcomes that occurred at any time during the hospital stay". |
| **Reznek et al. [37]** | Cox Proportional Hazards regression | patients who died in hospital | OR (95% CI) | 1.2 fold risk (1.03-1.36) | NA | Not available | "Non-ICU patients who died in Hospital had higher risk of having experienced longer boarding times. However, we did not observe a difference among ICU patients". |
| **Al-Khathaami et al. [38]** | Logistic regression | 0 - 0.75 hrs | OR (95% CI) | … | … | Age, sex, HTN*, diabetes, AF*, heart failure, previous stroke, hemorrhagic stroke, severity of stroke, onset to ED time, BT and ED wait time. | "There was no association between BT and the primary outcome. Only a history of heart failure and previous stroke, in addition to the patient having a moderate to severe stroke were associated with adverse events" |
|  |  | 0.76 - 1.42 hrs |  | 0.7 (0.37-1.44) | 0.36 |  |  |
|  |  | 1.43 - 2.97 hrs |  | 1.2 (0.64-2.36) | 0.51 |  |  |
|  |  | > 2.98 hrs |  | 0.5 (0.25-1.03) | 0.06 |  |  |
| **Hong et al. [39]** | Stepwise backward logistic regression | Quantitative (> 8 hrs) | OR (95% CI) | 3.4 (1.3-8.6) | 0.012 | NA | "We report an association between prolonged ED boarding stay and increased mortality in patients with necrotizing fasciitis". |

* APACHE score: acute physiology and chronic health evaluation score, SOFA: sequential organ failure assessment/sepsis, MAP: mean arterial pressure, TDD: time to decision to admit, CHF: congestive heart failure, COPD: chronic obstructive pulmonary disease, GI: gastro-intestinal, LOS: length of stay, BT: boarding time, OHCA: out-of-hospital cardiac arrest, ICU: intensive care unit, HTN: hypertension, AF: atrial fibrillation., ED: emergency department, OR: operating room, MICU: medical intensive care unit.
