# Supplementary material for: Association between boarding in the emergency department and in-hospital mortality: A systematic review
Source: PLoS One. 2020 Apr 15;15(4):e0231253. doi: 10.1371/journal.pone.0231253 (PMC7159217; doi:10.1371/journal.pone.0231253)
Supplement: S2 Table — (DOCX) [file pone.0231253.s002.docx]

| **Authors /reference** | **Title** | **Association of ED boarding with in-hospital mortality**  **Yes/ No** |
| --- | --- | --- |
| **Al-Qahtani et al. [28]** | The association of duration of boarding in the emergency room and the outcome of patients admitted to the intensive care unit | **Yes** |
| **Cha et al. [29]** | The impact of prolonged boarding of successfully resuscitated out-of-hospital cardiac arrest patients on survival-to-discharge rates. | **Yes** |
| **Chalfin et al. [30]** | Impact of delayed transfer of critically ill patients from the emergency department to the intensive care unit | **Yes** |
| **Hsieh et al. [31]** | Impact of delayed admission to intensive care units on patients with acute respiratory failure | **Yes** |
| **Gilligan et al. [32]** | The boarders in the emergency department (BED) study | **No** |
| **Junhasavasdikul et al. [33]** | Association between admission delay and adverse outcome of emergency medical patients | **No** |
| **Singer et al. [34]** | The association between length of emergency department boarding and mortality | **Yes** |
| **Augustin et al. [35]** | Impact of delayed admission to the intensive care unit from the emergency department upon sepsis outcomes and sepsis protocol compliance | **No** |
| **Lord et al. [36]** | Emergency department boarding and adverse hospitalization outcomes among patients admitted to a general medical service | **No** |
| **Reznek et al. [37]** | Mortality associated with emergency department boarding exposure: Are there differences between patients admitted to ICU and non-ICU settings | **Yes:** for non-ICU-admitted patients  **No:** for ICU-admitted patients |
| **Al-Khathaami et al. [38]** | The impact of “admit no bed” and long boarding times in the emergency department on stroke outcome | **No** |
| **Hong et al. [39]** | The effects of prolonged ED stay on outcome in patients with necrotizing fasciitis | **Yes** |

**Table 2. Results Summary:** A**ssociation between Emergency Department (ED) Boarding and In-Hospital Mortality.**
