# Supplementary material for: Association between boarding in the emergency department and in-hospital mortality: A systematic review
Source: PLoS One. 2020 Apr 15;15(4):e0231253. doi: 10.1371/journal.pone.0231253 (PMC7159217; doi:10.1371/journal.pone.0231253)
Supplement: S3 Table — (DOCX) [file pone.0231253.s003.docx]

**Table 3: Crude data observed in the control group non-exposed to boarding time and experimental group exposed to the boarding time over the cut-off reported in the selected studies.**

| Study | Boarding time cut-off (hours) | Study group (n) | Exposed (research group)  >Boarding time | | | Not exposed (control group)  <Boarding time | | | Odds ratio | CI (95%) | Comments |
| --- | --- | --- | --- | --- | --- | --- | --- | --- | --- | --- | --- |
|  |  |  | Total | Dead | Alive | Total | Dead | Alive |  |  |  |
| Al-Qahtani et al. [28] | 6 | 940 | 713 | 236 | 477 | 227 | 51 | 176 | 1.707 | 1.205  -  2.419 |  |
| Cha et al. [29] | 6 | 4,686 | 1267 | 974 | 293 | 3419 | 2386 | 1033 | 1.439 | 1.239  -  1.671 |  |
| Chalfin et al. [30] | 6 | 50,322 | 1036 | 180 | 856 | 49286 | 6358 | 42928 | 1.420 | 1.206  -  1.671 |  |
| Hsieh et al. [31] | 1 | 267 | 200 | 60 | 140 | 67 | 11 | 56 | 2.182 | 1.069  -  4.454 |  |
| Gilligan et al. [32] | Continuous  Mean 16.1  Range (0-161) | 13,357 | NA | NA | NA | NA | NA | NA | 0.998 | 0.983  –  1.013 | Calculated upper value of CI |
| Junhasavasdikul et al. [33] | Continuous  Lead Time | 381 | NA | NA | NA | NA | NA | NA | 0.970 | 0.932  –  1.01 |  |
| Singer et al. [34] | 2 | 41,256 | 20729 | 642 | 20087 | 20527 | 513 | 20014 | 1.247 | 1.108  -  1.403 |  |
| Augustin et al. [35] | 6 | 287 | 150 | 37 | 113 | 137 | 31 | 106 | 1.120 | 0.649  –  1.933 |  |
| Lord et al. [36] | 4 | 31,219 | 3978 | 1 | 3977 | 27,241 | 12 | 27,229 | 0.571 | 0.074  -  4.389 |  |
| Reznek et al. [37] | Continuous | 39,781 | 23 | NA | NA | NA | NA | NA | 1.2 | 1.03  -  1.398 | Calculated upper value of CI |
| Al-Khathaami et al. [38] | 0.75 | 300 | 213 | NA | NA | 75 | NA | NA | 0.7 | 0.37  -  1.324 | Calculated upper value of CI |
| Hong et al. [39] | 8 | 195 | 70 | 15 | 55 | 125 | 13 | 112 | 2.350 | 1.045  -  5.281 |  |
