# Supplementary material for: Association between boarding in the emergency department and in-hospital mortality: A systematic review
Source: PLoS One. 2020 Apr 15;15(4):e0231253. doi: 10.1371/journal.pone.0231253 (PMC7159217; doi:10.1371/journal.pone.0231253)
Supplement: S4 Table — (DOCX) [file pone.0231253.s004.docx]

**Table 4. Quality Assessment of the Studies (Newcastle-Ottawa Quality Assessment Scale).**

| **Authors/ reference** | **Selection** | | | | **Comparability of cohorts** | **Outcome** | | | **Total Score T** |
| --- | --- | --- | --- | --- | --- | --- | --- | --- | --- |
|  | **Representativeness of the exposed cohort** | **Selection of the nonexposed cohort** | **Ascertainment of exposure** | **Outcome of interest not present at study start** |  | **Assessment** | **Length of follow-up** | **Adequacy of follow-up of cohorts** |  |
| **Al-Qahtani et al. [28]** | * |  | * | * | * | * |  |  | 6 |
| **Cha et al. [29]** | * | * |  |  | * | * |  | * | 5 |
| **Chalfin et al. [30]** | * | * | * | * | * | * | * | * | 8 |
| **Hsieh et al. [31]** |  |  | * | * | * | * | * | * | 6 |
| **Gilligan et al. [32]** | * |  | * |  | * | * | * |  | 5 |
| **Junhasavasdikul et al. [33]** | * |  | * | * | * | * |  |  | 5 |
| **Singer et al. [34]** | * | * | * | * |  | * | * | * | 7 |
| **Augustin et al. [35]** |  | * |  | * | * | * | * |  | 5 |
| **Lord et al. [36]** |  | * | * | * | * | * |  | * | 6 |
| **Reznek et al. [37]** | * | * | * | * | * | * | * | * | 8 |
| **Al-Khathaami et al. [38]** | * |  | * | * | * | * |  |  | 5 |
| **Hong et al. [39]** | * | * | * |  | * | * |  | * | 6 |
