# Supplementary material for: Association between boarding in the emergency department and in-hospital mortality: A systematic review
Source: PLoS One. 2020 Apr 15;15(4):e0231253. doi: 10.1371/journal.pone.0231253 (PMC7159217; doi:10.1371/journal.pone.0231253)
Supplement: S5 Table — (DOCX) [file pone.0231253.s005.docx]

**Table 5. Evaluation of Risk of Bias.**

| **Authors/ reference** | 1. Inclusion /exclusion varies between groups | 2. Recruitment varies between groups | 3. Sample size sufficiently large | 4. Description of the intervention or exposure | 5. Outcome assessor blinded | 6. Valid and reliable measures | 7. Differential follow-up | 8. Impact of highloss to follow-up | 9. Missing outcomes | 10. Missing harm or adverse event data | 11. Results believable | 12. Balance of allocation between groups | 13. Confounding addressed appropriately |
| --- | --- | --- | --- | --- | --- | --- | --- | --- | --- | --- | --- | --- | --- |
| **Al-Qahtani et al. [28]** | **+** | **+** | ? | ? | ? | + | ? | ? | + | ? | + | ? | Part |
| **Cha et al. [29]** | **+** | ? | ? | ? | ? | + | + | ? | + | ? | + | ? | Part |
| **Chalfin et al. [30]** | **+** | + | + | ? | ? | + | ? | ? | + | ? | + | ? | Part |
| **Hsieh et al. [31]** | **+** | + | ? | ? | ? | ? | ? | ? | + | ? | + | ? | Part |
| **Gilligan et al. [32]** | **+** | ? | ? | ? | ? | ? | ? | ? | + | ? | + | ? | - |
| **Junhasavasdikul et al. [33]** | **+** | ? | ? | ? | ? | + | ? | ? | + | ? | + | ? | - |
| **Singer et al. [34]** | **+** | ? | ? | ? | ? | + | ? | ? | + | ? | + | ? | + |
| **Augustin et al. [35]** | **+** | ? | ? | ? | ? | + | ? | ? | + | ? | + | ? | Part |
| **Lord et al. [36]** | **+** | **+** | ? | ? | ? | + | ? | ? | + | ? | + | ? | + |
| **Reznek et al. [37]** | **+** | + | ? | + | ? | + | + | ? | + | ? | + | ? | Part |
| **Al-Khathaami et al. [38]** | **+** | ? | - | ? | ? | - | ? | ? | **+** | ? | **+** | ? | Part |
| **Hong et al. [39]** | **+** | **+** | **+** | ? | ? | ? | + | ? | **+** | ? | **+** | ? | Part |

+ Study met criteria, - Study did not meet criteria, ? Not applicable, Part: partially met criteria
