# Supplementary material for: Association between boarding in the emergency department and in-hospital mortality: A systematic review
Source: PLoS One. 2020 Apr 15;15(4):e0231253. doi: 10.1371/journal.pone.0231253 (PMC7159217; doi:10.1371/journal.pone.0231253)
Supplement: S1 Appendix — (DOCX) [file pone.0231253.s007.docx]

**S1 Appendix 1. Literature Search**

| **Database** | **Search String** | **Notes** | **Results** |
| --- | --- | --- | --- |
| **PubMed**  **Search Date:**  2018-11-16  **Coverage:**  1890- | (("Emergency Responders"[Mesh] OR "Emergency Medicine"[Mesh] OR "Evidence-Based Emergency Medicine"[Mesh] OR "Emergency Service, Hospital"[Mesh] OR "Emergency Medical Services"[Mesh] OR "Emergency Services, Psychiatric"[Mesh] OR "emergency room"[Title/Abstract] OR "emergency department"[Title/Abstract] OR "emergency ward"[Title/Abstract] OR "emergency patient"[Title/Abstract] OR "emergency patients"[Title/Abstract] OR “ED”[Title/Abstract] OR “ER"[Title/Abstract] OR “emergency medicine”[Title/Abstract] OR "emergency medical service"[Title/Abstract] OR "emergency medical services"[Title/Abstract] OR "emergency departments”[Title/Abstract] OR “emergency wards”[Title/Abstract] OR “emergency unit”[Title/Abstract] OR “emergency units”[Title/Abstract] OR “emergency rooms”[Title/Abstract] OR “emergency responders”[Title/Abstract] OR “emergency responder”[Title/Abstract]) AND ("Death"[Mesh] OR "Mortality"[Mesh] OR "Hospital Mortality"[Mesh] OR IHM[Title/Abstract] OR deaths[Title/Abstract] OR mortalities[Title/Abstract] OR death[Title/Abstract] OR mortality[Title/Abstract])AND (boarding[Title/Abstract] OR boarded[Title/Abstract] OR overcrowded[Title/Abstract] OR crowded[Title/Abstract] OR crowding[Title/Abstract] OR overcrowding[Title/Abstract] OR "Crowding"[Mesh] OR "Bed occupancy"[Mesh] OR "access blocks"[Title/Abstract] OR "access block"[Title/Abstract] OR "lead-time"[Title/Abstract] OR "admission delays”[Title/Abstract] OR "delayed admissions”[Title/Abstract] OR "patient admission" [Title/Abstract] OR "patient admissions"[Title/Abstract] OR "Patient Admission"[Mesh] OR "admission delay”[Title/Abstract] OR "bed occupancy”[Title/Abstract] OR “delayed transfer”[Title/Abstract] OR "delayed admission”[Title/Abstract] OR “bed management”[Title/Abstract])) | All terms searched in the fields for title and abstract (here marked with “Title/Abstract”) and in “MeSH” when available.  No filters or limitations applied.  **Notes:**  “Boarding time” is automatically searched with “boarding”. | **1451** |
| **Scopus**  **Search Date:**  2018-11-16  **Coverage:**  All publication years included | ((TITLE-ABS-KEY: "emergency room" OR "emergency department" OR "emergency ward" OR "emergency patient" OR "emergency patients" OR “ED” OR “ER" OR “emergency medicine” OR "emergency medical service" OR "emergency medical services" OR "emergency departments” OR “emergency wards” OR “emergency unit” OR “emergency units” OR “emergency rooms” OR “emergency responders” OR “emergency responder”) AND (TITLE-ABS-KEY: IHM OR deaths OR mortalities OR death OR mortality) AND (TITLE-ABS-KEY: boarding OR boarded OR overcrowded OR crowded OR crowding OR overcrowding OR "access blocks" OR "access block" OR "lead-time" OR "admission delays” OR "delayed admissions” OR "patient admission" OR "patient admissions" OR "admission delay” OR "bed occupancy” OR “delayed transfer” OR "delayed admission” OR “bed management”)) | All terms searched in the field “Article title, Abstract, Keywords” (here marked with “TITLE-ABS-KEY”).  No filters or limitations applied.  No thesaurus available. | **1286** |
| **Embase**  (Elsevier)  **Search Date:**  2018-11-16  **Coverage:**  1947- | (('emergency room':ab,ti OR 'emergency department':ab,ti OR 'emergency ward':ab,ti OR 'emergency patient':ab,ti OR 'emergency patients':ab,ti OR 'ed':ab,ti OR 'er':ab,ti OR 'emergency medicine':ab,ti OR 'emergency medical service':ab,ti OR 'emergency medical services':ab,ti OR 'emergency departments':ab,ti OR 'emergency wards':ab,ti OR 'emergency unit':ab,ti OR 'emergency units':ab,ti OR 'emergency rooms':ab,ti OR 'emergency responders':ab,ti OR 'emergency responder':ab,ti OR 'emergency medicine'/de OR 'psychiatric emergency service'/de OR 'evidence based emergency medicine'/de OR 'hospital emergency service'/de OR 'rescue personnel'/de) AND ('hospital admission'/de OR 'crowding (area)'/de OR 'hospital bed utilization'/de OR 'boarding':ab,ti OR 'boarded':ab,ti OR 'overcrowded':ab,ti OR 'crowded':ab,ti OR 'crowding':ab,ti OR 'overcrowding':ab,ti OR 'access blocks':ab,ti OR 'access block':ab,ti OR 'lead-time':ab,ti OR 'admission delays':ab,ti OR 'delayed admissions':ab,ti OR 'patient admission':ab,ti OR 'patient admissions':ab,ti OR 'admission delay':ab,ti OR 'bed occupancy':ab,ti OR 'delayed transfer':ab,ti OR 'delayed admission':ab,ti OR 'bed management':ab,ti) AND (ihm OR deaths OR mortalities OR death OR mortality OR 'hospital mortality'/de OR 'death'/de OR 'mortality'/de)) | All terms searched in the fields for “Title” and “Abstract” (here marked with “:ab,ti”) and in the “Emtree” (here marked with “/de”), when available.  **Filters or limitations applied:**  Source selected- “Embase only”.  **Thesaurus/subject headings variations compared to PubMed’s MeSH:**  “Emergency Medical Services” is referred to “Emergency Health Service” in the Emtree Not included.  "Emergency Service, Hospital" is referred to “Hospital Emergency Service” in the Emtree. Included.  “Emergency Responders” is referred to “Rescue Personnel” in the Emtree. Included.  "Emergency Services, Psychiatric" is referred to “Psychiatric Emergency Service” in the Emtree. Included.  “Patient admission” referees to “hospital admission” in the Emtree. Included.  “Bed occupancy" referees to “hospital bed utilization in the Emtree. Included. | **1990** |
| **Medline**  (WOS)  **Search Date:**  2018-11-16  **Coverage:** 1955- | ((MeSH HEADING: "Emergency Responders" OR MeSH HEADING: "Emergency Medicine" OR MeSH HEADING: "Evidence-Based Emergency Medicine" OR MeSH HEADING: "Emergency Service, Hospital" OR MeSH HEADING: "Emergency Medical Services" OR MeSH HEADING: "Emergency Services, Psychiatric" OR TOPIC: "emergency room" OR TOPIC: "emergency department" OR TOPIC: "emergency ward" OR TOPIC: "emergency patient" OR TOPIC: "emergency patients" OR TOPIC: “ED”OR TOPIC: “ER" OR TOPIC: “emergency medicine” OR TOPIC: "emergency medical service" OR TOPIC: "emergency medical services" OR TOPIC: "emergency departments” OR TOPIC: “emergency wards” OR TOPIC: “emergency unit” OR TOPIC: “emergency units” OR TOPIC: “emergency rooms” OR TOPIC: “emergency responders” OR TOPIC: “emergency responder”) AND (MeSH HEADING: "Death" OR MeSH HEADING: "Mortality” OR MeSH HEADING: "Hospital Mortality” OR TOPIC: IHM OR TOPIC: deaths OR TOPIC: mortalities OR TOPIC: death OR TOPIC: mortality) AND (TOPIC: boarding OR TOPIC: boarded OR TOPIC: overcrowded OR TOPIC: crowded OR TOPIC: crowding OR TOPIC: overcrowding OR MeSH HEADING: "Crowding" OR MeSH HEADING: "Bed occupancy" OR MeSH HEADING: "Patient Admission" OR TOPIC: "access blocks" OR TOPIC: "access block" OR TOPIC: "lead-time" OR TOPIC: "admission delays” OR TOPIC: "delayed admissions” OR TOPIC: "patient admission" OR TOPIC: "patient admissions" OR TOPIC: "admission delay” OR TOPIC: "bed occupancy” OR TOPIC: “delayed transfer” OR TOPIC: "delayed admission” OR TOPIC: “bed management”)) | All terms searched in the field “Topic” and in MeSH (here marked with “MeSH HEADING”), when available.  No filters or limitations applied.  **Thesaurus/subject headings variations compared to PubMed’s MeSH:** none. | **1538** |
| **Cochrane**  **Search Date:**  2018-11-16  **Coverage:**  All publication years included. | (("emergency room":ti,ab,kw OR "emergency department":ti,ab,kw OR "emergency ward":ti,ab,kw OR "emergency patient" :ti,ab,kw OR "emergency patients":ti,ab,kw OR “ED” :ti,ab,kw OR “ER":ti,ab,kw OR “emergency medicine” :ti,ab,kw OR "emergency medical service":ti,ab,kw OR "emergency medical services":ti,ab,kw OR "emergency departments” :ti,ab,kw OR “emergency wards” :ti,ab,kw OR “emergency unit” :ti,ab,kw OR “emergency units” :ti,ab,kw OR “emergency rooms” :ti,ab,kw OR “emergency responders” :ti,ab,kw OR “emergency responder”:ti,ab,kw OR "Emergency Responders":MeSH descriptor OR "Emergency Medicine":MeSH descriptor OR "Evidence-Based Emergency Medicine":MeSH descriptor OR "Emergency Service, Hospital":MeSH descriptor OR "Emergency Medical Services":MeSH descriptor OR "Emergency Services, Psychiatric":MeSH descriptor) AND (IHM:ti,ab,kw OR deaths:ti,ab,kw OR mortalities:ti,ab,kw OR death:ti,ab,kw OR mortality:ti,ab,kw OR "Death" OR "Mortality":MeSH descriptor OR "Hospital Mortality":MeSH descriptor) AND (boarding:ti,ab,kw OR boarded:ti,ab,kw OR overcrowded:ti,ab,kw OR crowded:ti,ab,kw OR crowding:ti,ab,kw OR overcrowding:ti,ab,kw OR "access blocks":ti,ab,kw OR "access block":ti,ab,kw OR "lead-time":ti,ab,kw OR "admission delays” :ti,ab,kw OR "delayed admissions” :ti,ab,kw OR "patient admission":ti,ab,kw OR "patient admissions":ti,ab,kw OR "admission delay” :ti,ab,kw OR "bed occupancy” :ti,ab,kw OR “delayed transfer” :ti,ab,kw OR "delayed admission” :ti,ab,kw OR “bed management”:ti,ab,kw OR "Crowding":MeSH descriptor OR "Bed occupancy":MeSH descriptor OR "Patient Admission":MeSH descriptor)) | All terms searched in the field “Article title”, “Abstract”, Keywords” (here marked with “:ti,ab,kw”), and in the MeSH (here marked with “MeSH descriptor”), when available.  No filters or limitations applied. All publication types included.  **Thesaurus/subject headings variations compared to PubMed’s MeSH:** none. | **93**  (3 reviews and 90 trials) |
| **CINAHL**  (EBSCO)  **Search Date:**  2018-11-16  **Coverage:**  1937- | ((AB (*"*emergency room" OR "emergency department" OR "emergency ward" OR "emergency patient" OR "emergency patients" OR “ED” OR “ER" OR “emergency medicine” OR "emergency medical service" OR "emergency medical services" OR "emergency departments” OR “emergency wards” OR “emergency unit” OR “emergency units” OR “emergency rooms” OR “emergency responders” OR “emergency responder” ) OR TI ("emergency room" OR "emergency department" OR "emergency ward" OR "emergency patient" OR "emergency patients" OR “ED” OR “ER" OR “emergency medicine” OR "emergency medical service" OR "emergency medical services" OR "emergency departments” OR “emergency wards” OR “emergency unit” OR “emergency units” OR “emergency rooms” OR “emergency responders” OR “emergency responder”) OR MH ("Emergency Services, Psychiatric" OR "Emergency Medical Services" OR "Emergency Medicine" OR "Emergency Service") AND (TI (boarding OR boarded OR overcrowded OR crowded OR crowding OR overcrowding OR "access blocks" OR "access block" OR "lead-time" OR "admission delays” OR "delayed admissions” OR "patient admission" OR "patient admissions" OR "admission delay” OR "bed occupancy” OR “delayed transfer” OR "delayed admission” OR “bed management”) OR AB (boarding OR boarded OR overcrowded OR crowded OR crowding OR overcrowding OR "access blocks" OR "access block" OR "lead-time" OR "admission delays” OR "delayed admissions” OR "patient admission" OR "patient admissions" OR "admission delay” OR "bed occupancy” OR “delayed transfer” OR "delayed admission” OR “bed management” ) OR MH ("Crowding" OR "Bed occupancy" OR "Patient Admission") AND (TI (IHM OR deaths OR mortalities OR death OR mortality) OR AB (IHM OR deaths OR mortalities OR death OR mortality) OR MH ("Hospital Mortality" OR MH "Death" OR MH "Mortality" )) | All terms searched in the fields for “Title” (here marked with “TI”), “Abstract” (here marked with “TI”) and in the thesaurus, “CINAHL Headings” (here marked with “MH”), when available.  **Thesaurus/subject headings variations compared to PubMed’s MeSH:**  **“**Emergency Service, Hospital” is referred to “Emergency service” in CINAHL Headings. Included.  “Evidence-Based Emergency Medicine” is not included in CINAHL Headings.  “Emergency Responders” is not available in CINAHL Headings. | **609** |
| **Web of Science**  (Core Collection)  **Search Date:**  2018-11-16  **Coverage:**  1955- | ((TOPIC:"emergency room" OR "emergency department" OR "emergency ward" OR "emergency patient" OR "emergency patients" OR “ED” OR “ER" OR “emergency medicine” OR "emergency medical service" OR "emergency medical services" OR "emergency departments” OR “emergency wards” OR “emergency unit” OR “emergency units” OR “emergency rooms” OR “emergency responders” OR “emergency responder”) AND (TOPIC: IHM OR deaths OR mortalities OR death OR mortality) AND (TOPIC: boarding OR boarded OR overcrowded OR crowded OR crowding OR overcrowding OR "access blocks" OR "access block" OR "lead-time" OR "admission delays” OR "delayed admissions” OR "patient admission" OR "patient admissions" OR "admission delay” OR "bed occupancy” OR “delayed transfer” OR "delayed admission” OR “bed management”)) | All terms searched in the field “Topic” (includes title, abstract and author supplied keywords).  No filters or limitations applied.  No thesaurus available in Web of Science. | **674** |
| **PsychInfo**  (OVID)  **Search Date:**  2018-11-16  **Coverage:**  Journal coverage from 1806- | ((TI"emergency room" OR TI "emergency department" OR TI "emergency ward" OR TI "emergency patient" OR TI "emergency patients" OR TI “ED” OR TI “ER" OR TI “emergency medicine” OR TI "emergency medical service" OR "emergency medical services" OR "emergency departments” OR TI “emergency wards” OR TI “emergency unit” OR TI “emergency units” OR TI “emergency rooms” OR TI “emergency responders” OR TI “emergency responder” OR AB "emergency room" OR "emergency department" OR "emergency ward" OR AB "emergency patient" OR AB "emergency patients" OR AB “ED” OR AB “ER" OR AB emergency medicine” OR AB "emergency medical service" OR AB "emergency medical services" OR AB "emergency departments” OR AB “emergency wards” OR AB “emergency unit” OR “emergency units” OR AB “emergency rooms” OR AB “emergency responders” OR AB “emergency responder” OR DE "Emergency Services") AND (TI IHM OR TI deaths OR TI mortalities OR TI death OR TI mortality OR AB IHM OR AB deaths OR AB mortalities OR AB death OR AB mortality OR DE "Death and Dying") AND TI boarding OR TI boarded OR TI overcrowded OR TI crowded OR TI crowding OR TI overcrowding OR TI "access blocks" OR TI "access block" OR TI "lead-time" OR TI "admission delays” OR TI "delayed admissions” OR TI "patient admission" OR TI "patient admissions" OR TI "admission delay” OR TI "bed occupancy” OR TI “delayed transfer” OR TI "delayed admission” OR TI “bed management” OR AB boarding OR AB boarded OR AB overcrowded OR AB crowded OR AB crowding OR AB overcrowding OR AB "access blocks" OR AB "access block" OR AB "lead-time" OR AB "admission delays” OR AB "delayed admissions” OR AB "patient admission" OR AB "patient admissions" OR AB "admission delay” OR AB "bed occupancy” OR AB “delayed transfer” OR AB "delayed admission” OR AB “bed management” OR DE “Hospital admission” OR DE “Crowding”)) | All terms searched in the fields for “Title” (here marked with “TI”), “Abstract” (here marked with “AB”) and in the “Thesaurus” (here marked with “DE”), when available.  No filters or limitations applied.  **Thesaurus/subject headings compared to PubMed’s MeSH:**  “Evidence-Based Emergency Medicine”, “Emergency Medicine”, “Emergency Medical Services”, “Emergency Responder” and “Emergency Service, Hospital” are referred to “Emergency Services” in the thesaurus. Included.  "Death", "Mortality", and "Hospital Mortality” are referred to “Death and Dying” in the thesaurus. Included.  “patient admission” referees to “hospital admission” in the thesaurus. Included.  “Bed occupancy” and "Emergency Services, Psychiatric” not included in PsychInfo’s thesaurus. | **52** |
| **Total number of references before de- duplication:** | | | **7693** |
| **Total number of references after de- duplication:** | | | **4321** |

**Updated search in PubMed** **2019-05-29**

| **Database** | **Search String** | **Notes** | **Results** |
| --- | --- | --- | --- |
| **PubMed**  **Search Date:**  2018-11-16 | (("Emergency Responders"[Mesh] OR "Emergency Medicine"[Mesh] OR "Evidence-Based Emergency Medicine"[Mesh] OR "Emergency Service, Hospital"[Mesh] OR "Emergency Medical Services"[Mesh] OR "Emergency Services, Psychiatric"[Mesh] OR "emergency room"[Title/Abstract] OR "emergency department"[Title/Abstract] OR "emergency ward"[Title/Abstract] OR "emergency patient"[Title/Abstract] OR "emergency patients"[Title/Abstract] OR “ED”[Title/Abstract] OR “ER"[Title/Abstract] OR “emergency medicine”[Title/Abstract] OR "emergency medical service"[Title/Abstract] OR "emergency medical services"[Title/Abstract] OR "emergency departments”[Title/Abstract] OR “emergency wards”[Title/Abstract] OR “emergency unit”[Title/Abstract] OR “emergency units”[Title/Abstract] OR “emergency rooms”[Title/Abstract] OR “emergency responders”[Title/Abstract] OR “emergency responder”[Title/Abstract]) AND ("Death"[Mesh] OR "Mortality"[Mesh] OR "Hospital Mortality"[Mesh] OR IHM[Title/Abstract] OR deaths[Title/Abstract] OR mortalities[Title/Abstract] OR death[Title/Abstract] OR mortality[Title/Abstract])AND (boarding[Title/Abstract] OR boarded[Title/Abstract] OR overcrowded[Title/Abstract] OR crowded[Title/Abstract] OR crowding[Title/Abstract] OR overcrowding[Title/Abstract] OR "Crowding"[Mesh] OR "Bed occupancy"[Mesh] OR "access blocks"[Title/Abstract] OR "access block"[Title/Abstract] OR "lead-time"[Title/Abstract] OR "admission delays”[Title/Abstract] OR "delayed admissions”[Title/Abstract] OR "patient admission" [Title/Abstract] OR "patient admissions"[Title/Abstract] OR "Patient Admission"[Mesh] OR "admission delay”[Title/Abstract] OR "bed occupancy”[Title/Abstract] OR “delayed transfer”[Title/Abstract] OR "delayed admission”[Title/Abstract] OR “bed management”[Title/Abstract])) | All terms searched in the fields for title and abstract (here marked with “Title/Abstract”) and in “MeSH” when available.  Publication year filter for 2018-11-16- 2019-05-29 applied.  **Notes:**  “Boarding time” is automatically searched with “boarding”. | **36**  (no duplicates detected within the search) |
